# Does hyperbaric oxygen therapy have the potential to improve salivary gland function in irradiated head and neck cancer patients?

**DOI:** 10.1186/2045-9912-3-15

**Published:** 2013-07-02

**Authors:** Tiffany Hadley, Cassaundra Song, Lauren Wells, Jennifer Lehnhardt, Marcy Wells Rogers, Jeffery Anderson, Michael Terry, Brian Novy, Takkin Lo

**Affiliations:** 1 Department of Anesthesiology, Loma Linda University Medical Center, 11234 Anderson St., Loma Linda, CA 92354, USA; 2 Department of Respiratory Care, Loma Linda University Medical Center, 11234 Anderson St., Loma Linda, CA 92354, USA; 3 Loma Linda School of Dentistry, 11092 Anderson St., Loma Linda, CA 92354, USA; 4 Loma Linda University, 11060 Anderson St., Loma Linda, CA 92350, USA; 5 Department of Respiratory Care, Loma Linda University Medical Center, 11234 Anderson St., Loma Linda, CA 92354, USA; 6 Department of Restorative Dentistry, Loma Linda University School of Dentistry, 11092 Anderson St., Loma Linda, CA 92354, USA; 7 Department of Medicine, Division of Pulmonary and Critical Care Medicine, Loma Linda University Medical Center, 11234 Anderson St., Loma Linda, CA 92354, USA

**Keywords:** Osteoradionecrosis, Xerostomia, Saliva, Hyperbaric Oxygen Therapy

## Abstract

Following radiotherapy, many patients with osteoradionecrosis suffer from xerostomia, thereby decreasing their quality of life. Patients can develop problems with speech, eating, increased dental caries, dysphagia, fractured dentition, chronic refractory osteomyelitis and osteoradionecrosis. Symptoms associated with salivary gland dysfunction can be severe enough that patients terminate the course of their radiotherapy prematurely due to the decrease in their quality of life. Currently, the only treatments available to patients are palliative. A definitive treatment has yet to be discovered. Head and neck cancers, which comprise 5% of overall cancer treatments, rank 8th most expensive to treat in the United States today. Hyperbaric oxygen is being considered for the therapy of radiated salivary glands because it has been shown to stimulate capillary angiogenesis and fibroplasia in radiation treated tissues. It has been hypothesized that salivary acinar cells undergo apoptosis following radiation therapy. The purpose of this paper is to discuss the mechanisms of salivary gland injury and evaluate whether hyperbaric oxygen therapy improves salivary gland function in patients who develop xerostomia and osteoradionecrosis following head and neck radiation.

## Background

Head and neck cancers account for approximately 5% of the overall cancers treated in the United States and ranked the 8^th^ most expensive cancer in the United States today [1]. There are five primary sites that make up this group of cancers: larynx, pharynx, oral cavity, salivary glands, and paranasal sinuses [2]. Of these patients who undergo standard head and neck radiotherapy, significant damage to the salivary glands can occur and result in hyposalivation and xerostomia, which is the condition of dry mouth caused by decreased salivation. In addition, hyposalivation is among the most widely recognized causes of dental caries, and oral discomfort, which includes oral sores, changes in taste, difficulty chewing, swallowing, and difficulty with speech [3]. This condition place patients at risk for dental caries and tooth decay because saliva normally bathes the oral cavity and acts as a clearing agent [4]. Xerostomia is one of the most common complications of head and neck irradiation, and essentially all patients that undergo radiotherapy will develop some form of xerostomia as a result of damage to their major and minor salivary glands [5]. End-stage complications of hyposalivation include fractured dentition, osteonecrosis, and chronic refractory osteomyelitis. Patients affected by salivary gland dysfunction often terminate their radiotherapy course prematurely because they become malnourished and experience a significant decrease in their quality of life [5]. Intensity-modulated radiation therapy as opposed to traditional radiation therapy, acupuncture, other masticatory or gustatory stimulatory therapies, administration of cytoprotective agents (i.e. amifostine), stimulation of residual tissue with cholinergic muscarinic agents (i.e. pilocarpine and bethanecol), and various lubricating agents are some options to aid with symptom control from xerostomia, but they are only supportive therapies that provide short-term alleviation [3]. A more permanent and preventive therapy is yet to be discovered to improve salivary gland function in these patients.

Although it has been suggested that there might be recovery of salivary gland function many years after radiation therapy, many have found there is very little recovery in patients who do not receive salivary gland sparing techniques; and in patients receiving concomitant chemotherapy the incidence of oral complications is increased [6]. Research demonstrates that head and neck radiation affect the major and minor salivary glands, and the effect on these glands contributes to radiation-induced temporary or permanent xerostomia [7]. In one study using high resolution-magnetic resonance imaging (HR-MRI) for tissue evaluation, there appeared to be adverse morphologic changes in the parotid gland as early as one to two weeks into the start of radiation therapy and at lower radiation doses than previously expected [8]. In addition, comparing serous and mucous-secreting glands, it has been shown that mucous-secreting glands are not necessarily more resistant to radiotherapy. There are no widely accepted methods to protect mucous-secreting glands because they are in the main treatment field for most head and neck cancers [9]. However, the major mucous glands are equally important to salivary flow rate during the unstimulated rest period, as are the major serous glands [10]. In one study, no significant difference was observed between the functional response of the serous (PAR), and the seromucous (SM)/mucous (SL) glands on exposure to ionizing irradiation [10]. In the first two weeks of radiotherapy most of the damage has already become manifest and an 80% reduction in salivary flow rate is observed, regardless of the type of gland [10].

## Discussion

Hyperbaric oxygen therapy (HBOT) has been shown to promote angiogenesis, stem cell recruitment and collagen synthesis by increasing oxygen tension in tissues, especially irradiated tissues [3,11,12]. HBOT has been used as a primary and adjunctive treatment for medical conditions ranging from infections to non-healing ischemic wounds. In head and neck irradiated patients, HBOT is specifically indicated for those patients within said population who have developed osteoradionecrosis (ORN), and for those who have developed other significant tissue damage that is non-healing. Studies have disproved the notion that the angiogenic properties brought about by HBOT increase the potential for recurrence, or induction of neoplastic tissue [13]. When sufficient capillary perfusion develops to eliminate oxygen gradients, excess or unnecessary angiogenesis is prevented, which is why perfusion only reaches about 75-85% of that of non-irradiated tissue [14]. Therefore, a protocol of 20 HBO treatments prior to surgery in irradiated tissue and 10 treatments post-surgery is most often used as it has been shown to decrease wound dehiscence, decrease rate of infection, and decrease wound healing delay that would lengthen the hospital stay for the patient [14].

Spontaneous and anecdotal reports of improvement in dry mouth at the Copenhagen University Hospital led to a pilot study evaluating the effect of HBOT on salivary flow rate in previously head and neck irradiated patients [3]. According to their data, there was a significant decrease in xerostomia as well as hyposalivation in the patients evaluated [3]. A subsequent study was conducted by a group of investigators to evaluate the effect HBOT has on salivary flow rate, pH, and salivary bacteria involved in head and neck irradiated patients. These results showed that HBOT may be able to decrease caries risk due to its beneficial effects on pH, bacterial load in the oral cavity, and salivary flow [14]. In addition, this study established that the amount of saliva needed to avoid clinical xerostomia is an unstimulated flow rate of at least 0.1-0.3 ml/min [15,16]. The limitation of these two studies is that they lack a control group.

In a study evaluating salivary glands with post-radiation dysfunction, an increase in the preservation of specialized tissues was demonstrated following HBOT [17]. The result of radiation creates what has been called a “3 H” effect on the tissue: hypovascular, hypocellular, and hypoxic [18]. When normal tissues are damaged they develop a central area of tissue injury, or hematoma, surrounded by normal tissue; the oxygen tension in the central area is *low*, whereas adjacent tissues have normal oxygen tension, creating a steep oxygen gradient over a short distance. This induces the necessary cellular factors to aggregate for capillary budding, and collagen synthesis needed for wound healing [19]. In irradiated tissue there is a largely diffuse pattern of tissue injury that creates a shallow oxygen gradient, which induces no physiochemical response; the body does not recognize the irradiated tissue as a wound, and development of a “3 H” pattern minimizes the natural tissue oxygen gradient [18]. Irradiated tissue therefore does not spontaneously re-vascularize, or become re-perfused, like other tissues because of the “unique physics and pattern of tumoricidal radiation delivery” that establishes this pattern of injury [14]. HBOT has been shown to induce angiogenesis in radiation damaged tissue by infusing the tissues with oxygen and temporarily simulating the steep oxygen gradient across the wounded tissue that mimics normal tissue damage; therefore, the same regulatory control for angiogenesis in normal tissues is applied to radiation damaged tissue [12]. HBOT stimulates capillary angiogenesis and fibroplasia in tissues radiated with a dose exceeding 5,000 cGy [12]. Studies show that HBOT enhances fibroblastic, osteoblastic, osteoclastic, and angioblastic activities in bones [3,17]. Healing of the wounded tissue occurs as a gradual process and it was found that the key time frames where the most significant improvements were seen after the 10th, 18th, and 20th hyperbaric oxygen exposures [14]. The optimal dosage for hyperbaric oxygen treatment is 2.0 to 2.5 ATA (atmosphere absolute) for 90 minutes duration, five days a week over a 20-session treatment course [12].

It is anticipated that if vascular supply to the irradiated salivary gland tissues can be restored, enabling tissue regeneration, near-normal to normal salivary flow can also be restored. Findings in patients undergoing HBOT for the treatment of ORN recently lead to a pilot study in this field that demonstrated that those patients who were treated with HBOT post-radiotherapy reported a decrease in xerostomia as well as a clinical increase in saliva production [3]. Aside from this, a limited number of studies have been conducted to evaluate the effects of HBO therapy on irradiated salivary gland tissues [15]. A major limitation of previous studies was that they lacked a control group. Further research in this area utilizing a control group and evaluating specific elements of gland function may be future targets for therapy.

Changes in the oral microbial flora have also been reported in patients who have received head and neck radiation therapy. Following radiotherapy, patients have been shown to prefer soft and carbohydrate-rich foods [20]. With decreased salivary flow rate, oral clearance of sugar is prolonged, which can amplify the rapid progression of radiation caries [15]. Many studies show that *S. mutans, Lactobacillus spp.,* and *C. albicans* can all increase following a physiologic change such as radiation therapy of the head and neck. *S. mutans* are the primary cause of bacteriological caries, and are found in high amounts in patients following radiation therapy. These bacteria metabolize sugars to produce acids capable of dissolving tooth enamel, which eventually becomes cavitated. *S. mutans* is thought to be a stable colonizer in the oral cavity, but when the oral cavity undergoes major physiologic changes such as xerostomia, changes to the oral microbial flora can often be found [20]. *Lactobacillus*, in addition to *S. mutans*, is a recognized etiologic agent of dental caries. *Lactobacillus* metabolizes sugars into lactic acid that sits on teeth and is capable of dissolving enamel, also resulting in cavitation. *Lactobacillus* is responsible for the high progression rate of existing caries. *C. albicans* is observed in infections occurring after radiation therapy and is the causative organism of candidiasis, or oral thrush [15].

In addition to changes in salivary flow, it is expected that the composition of whole saliva would be affected by radiation therapy and that compositional changes could also be evaluated for improvements in gland function. In one study, it was found that salivary flow rate, amylase activity, and protein content decreased following radiation therapy in the oral cavity of cancer patients [21]. Salivary amylase is one of the major protein components of parotid saliva, and if angiogenesis leads to a restoration of function in radiation-damaged acinar cells, salivary amylase should be measurably increased in whole saliva [21].

It has been shown that insulin growth factor-1 (IGF-1) injections following radiation therapy can gradually restore salivary gland function. In one study, total amylase content in irradiated glands was increased following intra-parotid IGF-1 injections, which demonstrates that an increase in function of the salivary acinar cells occurred [22]. IGF-1 is a potent activator of the threonine protein kinase Akt pathway in salivary acinar cells *in vitro,* and *in vivo*[23]. In a mouse model, apoptosis of acinar cells following irradiation was actually suppressed in those cells expressing the active form of Akt that were pre-treated with IGF-1 [23]. These results correspond with preservation of salivary gland function, and salivary flow rate [23].

In another study it was demonstrated that endothelial nitric oxide synthase (eNOS) is a modulator of angiogenesis, and that Akt activates eNOS via a phosphorylation pathway [24]. Endothelial cells, specifically in response to vascular endothelial growth factor-A (VEGF-A) or IGF-1, induce this phosphorylation pathway [23]. It was found that upon the addition of an inhibitor of Akt signaling, VEGF-A and IGF-1 concentrations were reduced, leading to reduced levels of phosphorylated eNOS, which in turn lead to the conclusion that Akt plays an important role in the regulation of angiogenesis [23]. It has been hypothesized that in a hyperoxic environment, VEGF-A activity is enhanced, which supports the use of HBOT as a means to achieve angiogenesis in a damaged tissue sample [25]. One study noted that acellular macrophage-conditioned media alone was sufficient to induce angiogenesis, and this was directly due to the presence of VEGF-A [26]. This is due to the fact that VEGF-A is significantly involved in the recruitment of other pro-angiogenic factors, including IGF-1 [26]. It is expected that HBOT will result in a clinically significant rise in both VEGF-A and IGF-1 salivary concentrations

Exploring the mechanisms of salivary gland injury may provide some insight on specific factors and pathways involved in salivary gland dysfunction. One theory concerning the mechanism of salivary gland damage is apoptosis of the salivary acinar cells. It has since been suggested that inhibition of the p53 apoptosis pathway might prove to be an effective method of protecting the salivary glands during therapeutic radiation treatment [27]. Radiation therapy has been shown to cause DNA damage, which activates the p53 pathway leading to cell death, cell cycle arrest and DNA repair [23]. Additionally, an *in vivo* study revealed that following head and neck irradiation, there was a marked increase in phosphorylated p53 and apoptosis in mouse parotid glands, and a resultant decrease in their salivary flow [27]. One review in the literature discussed that when cells are subjected to a hypoxic environment, or otherwise deficient in oxygen availability, they undergo adaptive responses to establish a new homeostasis in order to survive under such conditions [28]. If hypoxia lasts too long, or is too severe, the cells eventually undergo apoptosis [28]. This review discussed the possibility that the p53 pathway may be dependent on induction by and, competition with, hypoxia-inducible factor-1alpha (HIF-1 alpha) under prolonged hypoxic conditions. HIF-1alpha ordinarily helps to stabilize the transcription of target genes involved with the growth of blood vessels such as VEGF-A [28]. In cases of severe hypoxia, the drastic rise in HIF-1alpha levels that is produced under such conditions actually induces the stabilization of p53 [28]. Ordinarily, under hypoxic conditions, p53 levels fall as part of a protective mechanism to prevent apoptosis [28]. The resultant high levels of both factors create co-activator competition: while p53 stabilizes, HIF-1alpha activity decreases, leading to apoptosis [28]. Also, in cases of anoxia, a p53-dependent HIF-1alpha degradation pathway that leads to high levels of apoptosis was identified [28]. Sermeus mentioned that there are at least three different mechanisms used by p53 to inhibit angiogenesis. First, it increases the production of anti-angiogenic factors, such as thrombospondin-1. Second, p53 directly inhibits the HIF-1alpha pathway. Third, p53 transcriptionally represses genes encoding pro-angiogenic factors, including VEGF-A and basic fibroblast growth factor [28]. Perhaps one of the reasons why patients experience permanent damage to their salivary glands and marked xerostomia is due to the severe or prolonged hypoxic/anoxic environment caused by radiation therapy. Utilizing the advantages of HBOT, there is the potential for HBO to target specific points of insult and to modulate dysfunctional salivary gland tissue.

## Conclusion

Our study is anticipated to show the efficacy of HBOT in reducing xerostomia for head and neck cancer patients who have undergone radiation. We also intend to prove that HBOT will reduce costs associated with treating irradiated tissue. Annual cost of dental care incurred by head and neck cancer patients in the US has not been reviewed; however, as previously mentioned, head and neck cancers have been ranked the 8th most expensive cancers in the US today [1]. In addition to the millions spent on patients’ *medical* care (i.e. radiotherapy, chemotherapy, histographic sectioning, transfusions, surgical reconstruction, etc.), dental care costs are substantial in the US for this group and contribute to the detrimental effect to standard of living, especially since prevalence of patient dental insurance is low compared to medical insurance [1]. A UK study conducted in 2011 estimated that the dental costs of post-operative treatment for resected squamous cell carcinoma ranges from approximately £5,790 - £23,212 (approximately $9,218.87-$36,958.28), depending on the site of the primary lesion [29]. This included the cost of oral surgery, oral and maxillofacial surgery, palliative care, and pain management [29]. A US study published in 2012 reported a total annual health care spending for oral, oral pharyngeal and salivary gland cancers in their first year after diagnosis to be $79,151 for patients with commercial health insurance [2]. Patients receiving all three treatments (surgery, radiation, and chemotherapy) had the highest costs of care, from $96,520 in the Medicare population to $153,892 in the Commercial population [2]. If HBOT proves to be an effective treatment modality for resolution of hyposalivation, it may have the potential to significantly reduce medical and dental costs incurred for patients following tumor resection and radiation therapy. Most importantly, HBOT could possibly improve the quality of life for head and neck cancer patients by reducing the negative affects of radiation [30].

After reviewing the above principles of HBOT as well as mechanisms of salivary gland tissue injury from radiation, it would be clinically significant to perform this research study in order to explore possible ways of preserving and restoring salivary gland function. This prospective study proposal targets the specific angiogenic and anti-apoptotic factors that have the potential to demonstrate favorable clinical results. There is the expectation that evaluating the above theories may eventually provide information to determine if HBOT can be used as a prophylactic therapy for head and neck radiation therapy, or be integrated into the protocol for recovery for head and neck irradiated patients.

## Competing interests

The authors declare that they have no competing interests.

## Authors’ contributions

TH carried out the literature search and writing of the first and final draft of the manuscript. CS also performed a literature search, writing of the final draft and responding to peer reviewers. LW, JL, MWR, JA, MT, BN and TL wrote the initial study proposal, which serves as the basis to this manuscript. MT, BN and TL were staff and faculty advisors. All authors read and approved the final manuscript.
